# Association Between the Fukuda Stepping Test and Foulage Test Parameters in Healthy Adults and Patients With Ménière’s Disease

**DOI:** 10.7759/cureus.107722

**Published:** 2026-04-26

**Authors:** Toru Miwa, Teppei Kouga, Daichi Yamamoto, Tomohisa Yasuda

**Affiliations:** 1 Otolaryngology, Teikyo University Hospital, Mizonokuchi, Kawasaki, JPN; 2 Otolaryngology, Osaka Metropolitan University, Osaka, JPN; 3 Otolaryngology, Yasuda ENT Clinic, Tokyo, JPN

**Keywords:** dynamic posturography, foulage test, fukuda or unterberger stepping test, ménière’s disease, vestibular function

## Abstract

Background: The Foulage test is a stabilometer-based dynamic equilibrium assessment that enables quantitative evaluation of postural instability during stepping-like movement. However, its relationship with the conventional Fukuda stepping test (also known as the Unterberger stepping test) has not been fully clarified.

Objective: The objective of this study is to investigate the association between parameters obtained from the Fukuda stepping test and those obtained from the Foulage test in healthy adults and patients with Ménière’s disease during the interictal period.

Methods: This observational pilot study included 15 healthy adults and 15 patients with definite Ménière’s disease evaluated at the Department of Otolaryngology-Head and Neck Surgery, Osaka Metropolitan University, between July 1, 2023, and June 30, 2024. All participants underwent the Fukuda stepping test and the Foulage test under eyes-closed conditions. The migration distance and rotation angle were assessed in the Fukuda stepping test, whereas the FT value and theta value were assessed in the Foulage test. Because the aim of this study was to evaluate the magnitude of instability rather than directional laterality, absolute values were used for directional parameters. Group comparisons were performed using the unpaired t-test, and correlations were evaluated using Pearson’s correlation coefficient.

Results: Compared with healthy controls, patients with Ménière’s disease showed a significantly greater migration distance (2.02 ± 0.71 vs 0.29 ± 0.08, p < 0.001), rotation angle (22.0 ± 12.8° vs 5.0 ± 5.0°, p < 0.001), FT value (6.03 ± 1.51 vs 4.50 ± 0.60, p = 0.0017), and absolute theta value (15.46 ± 17.08° vs 1.87 ± 1.36°, p = 0.008). The migration distance showed a strong positive correlation with the FT value in both the healthy group (r = 0.872, p < 0.001) and the Ménière’s disease group (r = 0.824, p < 0.001). The rotation angle also showed a strong positive correlation with the absolute theta value in both the healthy group (r = 0.849, p < 0.001) and the Ménière’s disease group (r = 0.779, p < 0.001).

Conclusions: Foulage test parameters showed good correspondence with the major parameters of the Fukuda stepping test in both healthy adults and patients with Ménière’s disease during the interictal period. These findings suggest that the Foulage test may serve as a useful quantitative complementary assessment of dynamic equilibrium.

## Introduction

The Fukuda stepping test (also known as the Unterberger stepping test) is a classic and simple dynamic balance test used to evaluate vestibular asymmetry by observing the rotation angle, displacement angle, migration distance, and stepping trajectory during stepping with the eyes closed [1-3]. Although it remains widely used in clinical practice, it is primarily suited for assessing deviation and laterality and has limited ability to quantify postural instability itself. Previous studies have also reported limitations in its sensitivity, specificity, and reliability for identifying the affected side in vestibular dysfunction [1-3].

The Foulage test is a newer dynamic equilibrium test performed on a stabilometer. During the test, the participant alternately lifts the heels while keeping both metatarsal heads in contact with the platform, allowing quantitative assessment of dynamic balance based on the center-of-pressure trajectory [4-8]. This test can be performed with either the eyes open or closed and provides stabilometer-derived parameters such as the FT value, which reflects overall sway magnitude, and the theta value, which reflects rotational deviation [4]. Previous studies have suggested the usefulness of the Foulage test in both healthy individuals and patients with dizziness; however, its direct relationship with the conventional Fukuda stepping test has not been sufficiently investigated [5-7,9]. In particular, it remains unclear whether Foulage test parameters correspond to the conventional measures of displacement and rotation obtained from the Fukuda stepping test.

Therefore, in the present study, we performed both the Fukuda stepping test and the Foulage test under eyes-closed conditions in healthy adults and patients with Ménière’s disease during the interictal period. We hypothesized that the major parameters of the Foulage test would correlate with those of the Fukuda stepping test. The aim of this study was to determine whether the Foulage test corresponds to the conventional stepping test and whether it may serve as a useful quantitative complementary assessment of dynamic equilibrium.

## Materials and methods

Study design and participants

This observational pilot study included healthy adults and patients with definite Ménière’s disease who were evaluated at the Department of Otolaryngology, Osaka Metropolitan University, between July 1, 2023, and June 30, 2024. The study population consisted of 15 healthy participants and 15 patients with Ménière’s disease during the interictal period. The sample size was determined pragmatically based on the number of eligible participants who could be enrolled during the study period, and no formal a priori sample size calculation was performed.

Healthy participants were adults aged 20 to 89 years, including individuals with normal hearing, and were excluded if they had any apparent vestibular, neurological, or orthopedic disorder affecting activities of daily living. Patients with Ménière’s disease were required to meet the diagnostic criteria for definite Ménière’s disease according to the criteria of the Japan Society for Equilibrium Research and the Bárány Society [10,11]. Only patients who had remained free from vertigo attacks for at least three months were included. Patients with obvious neurological or orthopedic disorders affecting activities of daily living were excluded.

Written informed consent was obtained from all participants. The study was approved by the Ethics Committee of Osaka Metropolitan University (approval numbers: 2022-006 and 2023-086).

The healthy group had a mean age of 31.0 ± 7.3 years and consisted of 11 men and four women. Their mean height was 166 cm and mean body weight was 63 kg, and none had spontaneous nystagmus. The Ménière’s disease group had a mean age of 61.8 ± 18.4 years and consisted of seven men and eight women. Their mean height was 162 cm and mean body weight was 55 kg, and spontaneous nystagmus was observed in five patients during the interictal period.

Test procedures

All participants underwent both the Fukuda stepping test and the Foulage test under eyes-closed conditions. In the Fukuda stepping test, participants were instructed to step in place for 50 steps with both arms extended forward. No time limit was imposed. The migration distance and rotation angle were selected as the main outcome measures [1-3]. The migration distance was manually measured as the straight-line distance from the starting point to the final position using a measuring tape, and the rotation angle was manually measured using a protractor.

In the Foulage test, measurements were obtained according to the previously reported protocol [6]. The test was performed using a stabilometer (GW5000, Anima Co., Ltd., Tokyo, Japan). Participants alternately lifted their heels while keeping both metatarsal heads in contact with the platform. The FT value and absolute theta value were used as the main parameters. The FT value was derived from trajectory length and outer peripheral area and was considered to reflect the overall magnitude of sway, whereas the absolute theta value reflected the magnitude of rotational deviation.

Each participant performed three trials for both tests, and the mean values of the three trials were used for analysis.

Some parameters could take positive or negative values depending on direction. Because the purpose of this study was to evaluate the magnitude of dynamic instability rather than directional laterality, absolute values were used for directional parameters in the analysis. This approach eliminated direction dependence and allowed the analysis to focus on quantitative instability.

Statistical analysis

Continuous variables are presented as mean ± standard deviation, and categorical variables are presented as the number of patients (percentage). Normality of continuous variables was assessed using the Shapiro-Wilk test. Group comparisons between healthy participants and patients with Ménière’s disease were primarily performed using the unpaired t-test for continuous variables and Fisher’s exact test for categorical variables. Because of the small sample size and the use of absolute values for directional parameters, supplementary nonparametric analyses were also performed using the Mann-Whitney U test for group comparisons and Spearman’s rank correlation coefficient for correlation analyses. Within each group, Pearson’s correlation coefficient was used to examine the relationship between the migration distance in the Fukuda stepping test and FT value in the Foulage test, as well as the relationship between the rotation angle in the Fukuda stepping test and the absolute theta value in the Foulage test. A p-value < 0.05 was considered statistically significant. Because of the exploratory design and small sample size, no multivariable adjustment was performed, and the findings should be interpreted as hypothesis-generating rather than definitive.

## Results

Baseline characteristics of the study participants

Baseline characteristics of the study participants are summarized in Table 1. The mean age was significantly higher in the Ménière’s disease group than in the healthy group (61.8 ± 18.4 vs 31.0 ± 7.4 years, unpaired t-test, t = 6.01, df = 28, p < 0.001). Spontaneous nystagmus was observed in five patients (33.3%) in the Ménière’s disease group, whereas no spontaneous nystagmus was observed in the healthy group (0/15 [0%], Fisher’s exact test, p = 0.042).

**Table 1:** Baseline characteristics of the study participants Values are presented as mean ± standard deviation or number of patients (percentage). Continuous variables were compared using the unpaired t-test, and categorical variables were compared using Fisher’s exact test. A p-value < 0.05 was considered statistically significant.

	Healthy (n=15)	Ménière’s disease (n=15)	p-value
Age, years	31.0 ± 7.4	61.8 ± 18.4	<0.001
Sex, n (%)	11 (73.3%) /4 (26.7%)	7 (46.6%) /8 (53.4%)	0.265
Height, cm	166.3 ± 6.6	162.1 ± 8.8	0.146
Weight, kg	63.5 ± 8.6	55.8 ± 14.4	0.088
Spontaneous Nystagmus, n (%)	0 (0%)	5 (33.3%)	0.042

Comparison of the Fukuda stepping test and Foulage test parameters between groups

Comparisons of the Fukuda stepping test and Foulage test parameters are shown in Table 2. The migration distance in the Fukuda stepping test was significantly greater in the Ménière’s disease group than in the healthy group (2.02 ± 0.71 vs 0.29 ± 0.08 m, unpaired t-test, t = 9.38, df = 28, p < 0.001). The rotation angle was also significantly greater in the Ménière’s disease group (22.0 ± 12.8° vs 5.0 ± 5.0°, unpaired t-test, t = 4.79, df = 28, p < 0.001).

**Table 2:** Comparison of the Fukuda stepping test and Foulage test parameters between healthy participants and patients with Ménière’s disease Values are presented as mean ± standard deviation. Group comparisons were primarily performed using the unpaired t-test. Supplementary nonparametric analyses were performed using the Mann-Whitney U test. A p-value < 0.05 was considered statistically significant.

	Healthy (n=15)	Ménière’s disease (n=15)	p-value
Migration distance, m	0.29 ± 0.08	2.02 ± 0.71	<0.001
Rotation angle, degrees	5.0 ± 5.0	22.0 ± 12.8	<0.001
FT value	4.50 ± 0.60	6.03 ± 1.51	0.0017
|θ|, degrees	1.87 ± 1.36	15.46 ± 17.08	0.0047

In the Foulage test, the FT value was significantly higher in the Ménière’s disease group than in the healthy group (6.03 ± 1.51 vs 4.50 ± 0.60, unpaired t-test, t = 3.67, df = 28, p = 0.0010). Similarly, the absolute theta value was significantly higher in the Ménière’s disease group (15.46 ± 17.08° vs 1.87 ± 1.36°, unpaired t-test, t = 3.07, df = 28, p = 0.0047).

Normality testing and supplementary nonparametric analyses

The results of normality testing and supplementary nonparametric analyses are summarized in Table 3. Shapiro-Wilk testing showed that several variables, particularly migration distance, rotation angle, and absolute theta value in the healthy group, as well as the absolute theta value in the Ménière’s disease group, deviated from normality. Because of the small sample size and the use of absolute values for directional parameters, supplementary nonparametric analyses were performed.

**Table 3:** Normality testing and supplementary nonparametric analyses Normality of continuous variables was assessed using the Shapiro-Wilk test. Supplementary nonparametric group comparisons were performed using the Mann-Whitney U test, and supplementary nonparametric correlations were assessed using Spearman’s rank correlation coefficient. A p-value < 0.05 was considered statistically significant.

Analysis	Variable	Healthy	Ménière’s disease
Shapiro-Wilk test	Migration distance	W = 0.819, p = 0.006	W = 0.970, p = 0.857
Shapiro-Wilk test	Rotation angle	W = 0.845, p = 0.015	W = 0.929, p = 0.264
Shapiro-Wilk test	FT value	W = 0.890, p = 0.066	W = 0.952, p = 0.557
Shapiro-Wilk test	|θ|	W = 0.861, p = 0.025	W = 0.757, p = 0.001
Mann-Whitney U test	Migration distance	-	U = 225.0, p < 0.001
Mann-Whitney U test	Rotation angle	-	U = 205.0, p < 0.001
Mann-Whitney U test	FT value	-	U = 196.5, p < 0.001
Mann-Whitney U test	|θ|	-	U = 203.5, p < 0.001
Spearman correlation	Migration distance vs FT value	ρ = 0.808, p < 0.001	ρ = 0.818, p < 0.001
Spearman correlation	Rotation angle vs |θ|	ρ = 0.851, p < 0.001	ρ = 0.649, p = 0.0088

The supplementary Mann-Whitney U test showed results consistent with the primary parametric analyses for all between-group comparisons. In addition, supplementary Spearman correlation analyses showed positive associations consistent with the primary Pearson correlation analyses for both the migration distance versus FT value and rotation angle versus absolute theta value in each group.

Relationship between the migration distance and FT value

**Figure 1:**
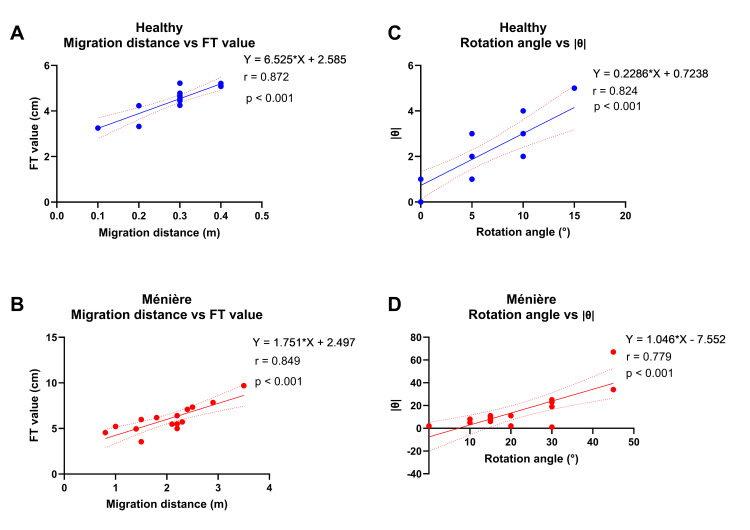
Correlations between the Fukuda stepping test and Foulage test parameters in healthy participants and patients with Ménière’s disease (A) Correlation between migration distance and FT value in healthy participants. (B) Correlation between migration distance and FT value in patients with Ménière’s disease. (C) Correlation between rotation angle and absolute theta value in healthy participants. (D) Correlation between rotation angle and absolute theta value in patients with Ménière’s disease. Correlations were evaluated using both Pearson’s correlation coefficient and Spearman’s rank correlation coefficient. The corresponding correlation coefficients and p-values are reported in the text. A p-value < 0.05 was considered statistically significant.

In the healthy group, the migration distance showed a strong positive correlation with the FT value (Figure 1A, Pearson’s r = 0.872, p < 0.001). A similarly strong positive correlation was observed in the Ménière’s disease group (Figure 1B, Pearson’s r = 0.824, p < 0.001). Supplementary Spearman correlation analyses also showed consistent positive associations in the healthy group (ρ = 0.808, p < 0.001) and in the Ménière’s disease group (ρ = 0.818, p < 0.001).

These findings indicate that the magnitude of displacement observed in the Fukuda stepping test corresponds closely to the instability index represented by the FT value in the Foulage test.

Relationship between the rotation angle and absolute theta value

In the healthy group, the rotation angle showed a strong positive correlation with the absolute theta value (Figure 1C, Pearson’s r = 0.843, p < 0.001). A strong positive correlation was also found in the Ménière’s disease group (Figure 1D, Pearson’s r = 0.779, p < 0.001). Supplementary Spearman correlation analyses also showed consistent positive associations in the healthy group (ρ = 0.851, p < 0.001) and in the Ménière’s disease group (ρ = 0.649, p = 0.0088).

These findings suggest that the rotational component of the Fukuda stepping test is well reflected by the absolute theta value in the Foulage test.

## Discussion

In this study, we examined the relationship between the Fukuda stepping test and the Foulage test in healthy adults and patients with Ménière’s disease during the interictal period. Patients with Ménière’s disease showed significantly greater migration distance, rotation angle, FT value, and absolute theta value than healthy controls. These findings suggest that both deviation and instability are increased in patients with Ménière’s disease and can be detected by both conventional stepping assessment and stabilometer-based dynamic equilibrium testing. This interpretation is consistent with previous reports showing that posturography can provide objective information on postural control impairment in Ménière’s disease [12,13]. Although several variables deviated from normality, supplementary nonparametric analyses yielded results consistent with the primary parametric analyses, supporting the robustness of the observed associations.

A major finding of this study was the strong correlation between the migration distance and FT value in both groups. Migration distance in the Fukuda stepping test is a relatively simple marker of whole-body displacement during stepping [1-3], whereas the FT value integrates stabilometric information derived from trajectory length and sway area [6,7]. The strong correspondence between these variables suggests that the Foulage test may quantitatively reflect instability that has traditionally been assessed only qualitatively or semi-quantitatively in the conventional stepping test.

Another important finding was the strong correlation between the rotation angle and the absolute theta value in both groups. This supports the view that the Foulage test can also quantify the degree of rotational deviation measured by the Fukuda stepping test. Given that the conventional stepping test is simple and widely used but has limited ability to quantify instability and some limitations in side localization [1-3], the Foulage test may be better positioned as a quantitative complementary test rather than a direct replacement.

This study has several limitations. First, this was a small exploratory pilot study, and no formal sample size calculation was performed. Therefore, the findings should be regarded as hypothesis-generating rather than definitive. Second, the Ménière’s disease group was significantly older than the healthy group, and age-related decline in postural control may have contributed to the observed group differences [14]. In addition, no age-adjusted multivariable analysis was performed; therefore, the between-group comparisons should be interpreted with caution. Third, only patients with Ménière’s disease during the interictal period were included, and the findings cannot necessarily be generalized to other vestibular disorders. Finally, because directional parameters were converted to absolute values in order to focus on the magnitude of instability, the present study did not evaluate lateralization accuracy. Future studies with age-matched controls, larger cohorts, and disease-specific subgroup analyses should examine directional accuracy and the potential lateralization value of the Foulage test in comparison with the Fukuda stepping test.

## Conclusions

Foulage test parameters showed good correspondence with the major parameters of the Fukuda stepping test in both healthy adults and patients with Ménière’s disease during the interictal period. In particular, the migration distance was strongly associated with the FT value, and the rotation angle was strongly associated with the absolute theta value. These findings are hypothesis-generating and suggest that the Foulage test may serve as a useful quantitative complementary assessment of dynamic equilibrium.
